# An Intermolecular π-Stacking Interaction Drives Conformational Changes Necessary to β-Barrel Formation in a Pore-Forming Toxin

**DOI:** 10.1128/mBio.01017-19

**Published:** 2019-07-02

**Authors:** Joshua R. Burns, Craig J. Morton, Michael W. Parker, Rodney K. Tweten

**Affiliations:** a Department of Microbiology and Immunology, University of Oklahoma Health Sciences Center, Oklahoma City, Oklahoma, USA; b Department of Biochemistry and Molecular Biology, Bio21 Molecular Science and Biotechnology Institute, The University of Melbourne, Parkville, Victoria, Australia; c St. Vincent’s Institute of Medical Research, Fitzroy, Victoria, Australia; University of Pittsburgh School of Medicine

**Keywords:** β-barrel, cholesterol-dependent cytolysin, membrane attack complex, oligomer, perforin

## Abstract

The crystal structures of the soluble monomers of the pore-forming cholesterol-dependent cytolysins (CDCs) contain two α-helical bundles that flank a twisted core β-sheet. This protein fold is the hallmark of the CDCs, as well as of the membrane attack complex/perforin immune defense proteins and the stonefish toxins. To form the β-barrel pore, a core β-sheet is flattened to align the membrane-spanning β-hairpins. Concomitantly with this conformational change, the two α-helical bundles that flank the core β-sheet break their restraining contacts and refold into two membrane-spanning β-hairpins of the β-barrel pore. The studies herein show that in the monomer structure of the archetype CDC perfringolysin O (PFO), a conserved Met-Met-Phe triad simultaneously contributes to maintaining the twist in this core β-sheet, as well as restricting the α-helical–to–β-strand transition necessary to form one of two membrane-spanning β-hairpins. A previously identified intermolecular π-stacking interaction is now shown to disrupt the interactions mediated by this conserved triad. This is required to establish the subsequent intermolecular electrostatic interaction, which has previously been shown to drive the final conformational changes necessary to form the β-barrel pore. Hence, these studies show that the intermolecular π-stacking and electrostatic interactions work in tandem to flatten the core β-sheet and initiate the α-helical–to–β-strand transitions to form the β-barrel pore.

## Abstract (precis)

A unique feature of the CDC/MACPF/SNTX (cholesterol-dependent cytolysin/membrane attack complex perforin/stonefish toxin) superfamily of pore-forming toxins is that the β-strands that comprise the β-barrel pore are derived from a pair of α-helical bundles. These studies reveal the molecular basis by which the formation of intermolecular interactions within the prepore complex drive the disruption of intramolecular interactions within each monomer of the prepore to trigger the α-helical–to–β-strand transition and formation of the β-barrel pore.

## INTRODUCTION

**FIG 1:**
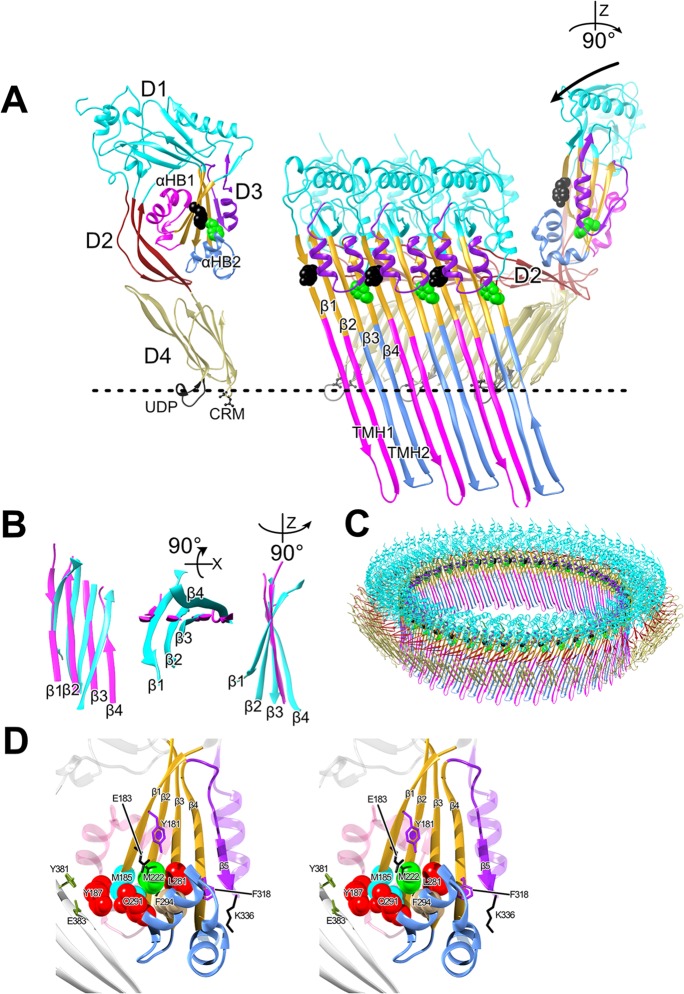
PFO structure and mechanism. (A) α-Carbon backbone traces of the crystal structure of the soluble PFO monomer (16) (left panel) and 3 PFO monomers from the pore complex model of PFO (right panel) shown in panel C, which is modeled on the 4.5-Å cryoEM structure of the closely related CDC pneumolysin (20). In the right panel, domain 4 of the soluble monomer structure was aligned with domain 4 of the monomer from the pore to show the ratchet-like lowering via domain 2 of domains 1 and 3 as they move ∼40-Å closer to the membrane (7, 20, 32). The vertical collapse of domain 3 toward the membrane is necessary so that the transmembrane β-hairpins 1 and 2 (TMH1 and -2, magenta and light blue, respectively), which form the β-barrel pore, can cross the membrane bilayer (3, 32) at a 20° tilt (10). Upon prepore-to-pore transition, α-helical bundles 1 and 2 (αHB1 and -2) refold into the membrane-spanning β-hairpins TMH1 and -2 to form the β-barrel pore (2, 3). (B) Overlay of the domain 3 core β-sheets from the soluble PFO monomer structure (16) (cyan) and membrane-inserted PFO monomer from the PLY-based pore model (magenta) showing the flattening of the twist in the β-sheet upon the transition to the pore. Shown left to right are the core β-sheet structures viewed from the interior of the oligomeric complex (a bottom-up view and a side view). (C) PFO pore modeled on the cryoEM-derived 4.5-Å resolution pneumolysin pore (20). (D) Stereo representation of the core β-sheet (gold) and αHBs of domain 3. Shown are the locations of the residues in this study. The dashed line in panels A and C shows the approximate location of the upper surface of the membrane. D1 to D4, domains 1 to 4; CRM, cholesterol recognition/binding motif; UDP; conserved undecapeptide.

A common feature shared among most β-barrel pore-forming toxins (βPFTs) is the formation of a prepore intermediate wherein membrane-bound monomers oligomerize into a supramolecular ring-shaped complex prior to the assembly and insertion of the β-barrel pore (1–3). Prepore intermediates that form both small and large β-barrel pores have been identified in βPFTs (1, 3–6). For the cholesterol-dependent cytolysins (CDCs), which form a large pore complex comprised of 35 to 40 monomers, each monomer within the prepore oligomer unfurls two α-helical bundles (αHB1 and αHB2) (Fig. 1) that refold into two amphipathic membrane-spanning β-hairpins (TMH1 and TMH2), which insert into the membrane bilayer to form the large β-barrel pore (2, 3, 7–11).

A thorough understanding of the prepore-to-pore transition remains elusive due to its fleeting existence, as upon prepore completion, it rapidly converts to the pore (12). However, a number of mutants that trap the archetype CDC, perfringolysin O (PFO), in various states of prepore assembly have been identified (8, 9, 13, 14). Two key intermolecular interactions that are necessary to the formation of the PFO pore is an aromatic π-stacking interaction and an electrostatic interaction. The loss of either interaction traps PFO in a prepore state (8, 9, 14). The formation of the electrostatic interaction disrupts a water network that stabilizes the interface between αHB1 and domains 1 and 2 (Fig. 1) (15). The disruption of this interface allows the transition of αHB1 and αHB2 into the transmembrane β-hairpins TMH1 and TMH2, which form the β-barrel pore, in each monomer of the prepore. In prepore-trapped mutants where the electrostatic interaction has been eliminated, full pore-forming activity can be restored by the introduction of a mutation at the domain 3-domain 1 and 2 interface that disrupts this water network (14, 15). Thus, the energy supplied by the electrostatic interaction drives the disruption of the final stage of the prepore-to-pore conversion, wherein the final contacts between αHB1 and domains 1 and 2 are broken (Fig. 1A).

We previously hypothesized that the intermolecular π-stacking interaction between residues Y181 in β-strand 1 of one monomer and F318 in β-strand 4 of a second monomer (Fig. 1) maintained the correct intermolecular register between juxtaposed membrane-spanning β-hairpins that form the β-barrel pore (9). We have now identified several point mutations that restore pore-forming activity to mutants lacking the π-stacking interaction. These studies reveal that the π-stacking interaction is apparently unnecessary for β-strand alignment, as pore formation can proceed without the formation of this interaction. Instead, our studies show that the π-stacking interaction drives the disruption of interactions between αHB2 and the domain 3 core β-sheet (Fig. 1). We further show that this action is necessary to establish the subsequent electrostatic interaction that drives the disruption of the final contacts between αHB1 with domains 1 and 2 (14). Hence, the π-stacking and electrostatic interactions work in tandem to drive structure changes in domain 3, which are required to refold the αHBs into the extended transmembrane hairpins (TMHs) that form the β-barrel pore.

## RESULTS

The residues of β-strand 1, which is one of four β-strands that form the core β-sheet of domain 3 (Fig. 1D), play key roles in the mechanism of PFO. Previous analyses of β-strand 1 residues have shown that it is highly sensitive to mutation, resulting in PFO protein variants unable to complete the prepore-to-pore transition (8, 9, 14). Two residues on the same side of β-strand 1, Y181 and E183 (Fig. 1D), form critical intermolecular interactions necessary for the prepore-to-pore conversion. Y181 forms a π-stacking interaction with F318 in β-strand 4 of an adjacent monomer within the prepore complex (8, 9) whereas E183 forms an intermolecular electrostatic interaction with K336, which is located at the tip of β5 (14). The loss of either interaction traps PFO in a prepore complex, which abrogates pore-forming activity (8, 9, 14). In sharp contrast to these mutations, the mutation of β-strand 1 residue M185, which is the next residue on the same surface of β-strand 1 as Y181 and E183, significantly increased the specific activity of PFO (Table 1), which prompted its further characterization.

**TABLE 1:** Specific activities of PFO and PFO variants^*a*^

Toxin	% of WT activity	*T_m_* (°C)
PFO	100	49.8
M185A mutant	349	47.2
E183A mutant	ND	48.2
Y181A mutant	ND	47.3
F318A mutant	ND	46.8
Q291A mutant	220	48.9
M222A mutant	229	46.8
N197W mutant	446	45.3
Y187A mutant	338	46.6
L281A mutant	110	46.1
F294A mutant	622	42.4
M185A:Y181A mutant	114	42.7
M185A:F318A mutant	65	48.1
M185A:E183A mutant	44	47.9
M222A:Y181A mutant	160	43.4
Y187A:Y181A mutant	ND	46.6
Q291A:Y181A mutant	ND	46.1
L281A:Y181A mutant	ND	45.0
F294A:Y181A mutant	625	41.7
N197W:Y181A mutant	ND	42.9
E183A:Y181A mutant	ND	47.6
M185A:E183A:Y181A mutant	ND	42.5

a The percent pore-forming activities of PFO and PFO variants are shown. The specific activity of pore formation was determined by marker (carboxyfluorescein [CF]) release from cholesterol-rich liposomes (EC_50_, effective concentration of toxin for the release of 50% of the CF from the liposomes). ND, not determined since the pore-forming activity did not approach 100% release at the highest concentration used. The *T_m_* data are the average of results from 4 experiments, with a standard error of < 0.3°C. Pore-forming activity data are means from at least 3 experiments, with a standard error of <0.5%. The shaded mutants and values are those that restore pore-forming activity in mutants that lack the intermolecular π-stacking interaction (e.g., the Y181A or F318A mutant).

### Mutation of M185 restores pore-forming activity in a mutant lacking the aromatic π-stacking interaction.

We previously hypothesized that the intermolecular π-stacking interaction between PFO β-strand 1 residue Y181 and β-strand 4 residue F318 was required to establish the correct intermolecular register between domain 3 transmembrane β-strands 1 and 4 as αHB1 and αHB2 refolded into TMH1 and TMH2 (9). However, when M185A was combined with prepore-trapped Y181A or F318A mutants, pore-forming activity was restored to wild-type (WT), or greater, levels (Table 1), suggesting that the intermolecular π-stacking interaction instead drives conformational changes within domain 3 necessary for pore formation. These data also suggest that the Y181-F318 π-stacking interaction is not required for the alignment of β-strands 1 and 4 of adjacent monomers during prepore assembly, as we previously suggested (9), since the β-barrel assembles in the absence of the π-stacking interaction in the M185A:Y181A and M185A:F318A mutants.

### Arrhenius activation energy for pore formation by PFO and its derivatives.

**FIG 2:**
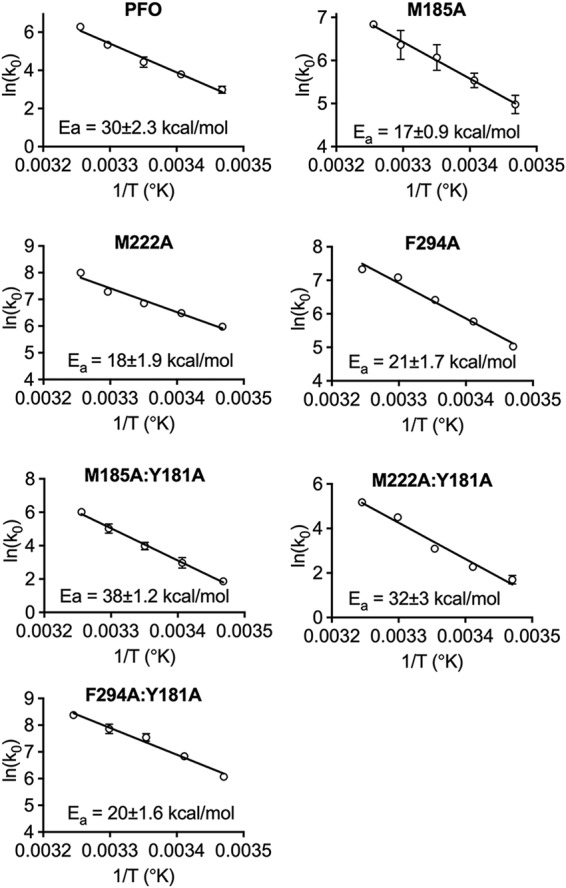
Arrhenius activation energies (*E_a_*s) of PFO and PFO variants. The initial velocities (*k*_0_s) of pore formation for PFO and its derivatives were determined by injecting the toxins directly into a stirred cuvette containing CF liposomes maintained at temperatures (T) from 15 to 35°C. The initial velocities (*k*_0_s) of pore formation were used to calculate the *E_a_*. All experiments were performed in triplicate. Standard error bars for all data points are included, although many do not show because they are smaller than the symbols.

The ability of M185A to restore activity to the Y181A and F318A mutants suggests that the π-stacking interaction disrupts M185-mediated interactions that are necessarily broken to facilitate the prepore-to-pore transition. If true, then the loss of these contacts in M185A would likely lower the transition state energy of pore formation, which would be reflected in the lower Arrhenius activation energy (*E_a_*) of pore formation. The *E_a_* of pore formation for PFO and derivatives was determined (Fig. 2). The *E_a_* for PFO was calculated to be 30 kcal/mol, similar to previous values (14, 15) (Fig. 2). The M185A mutation substantially reduced the *E_a_* for pore formation to 17 kcal/mol (Fig. 2). The pore-forming activity of the Y181A variant is not detectable at temperatures less than 35°C, and so its *E_a_* cannot be determined. However, when M185A and Y181A mutations were combined in PFO (M185A:Y181A), pore-forming activity was restored (Table 1) and its *E_a_* was determined to be 38 kcal/mol (Fig. 2). Hence, the M185A mutation restores pore-forming activity to mutants lacking the π-stacking interaction, presumably by lowering the *E_a_* into a range that allows other factors to drive the prepore-to-pore transition.

### Mutation of M185 partially restores pore-forming activity to PFO that lacks the E183-K336 electrostatic interaction.

The formation of an intermolecular electrostatic interaction between E183 and K336, in addition to the aromatic π-stacking interaction, is necessary to drive the prepore-to-pore transition. This interaction disrupts the final contacts between αHB1 and domains 1 and 2, thereby allowing the refolding of αHB1 into TMH1 (14, 15). Loss of this interaction through mutation of E183 or K336 to alanine (or other residues) also prevents pore formation by trapping PFO in an inactive prepore state (14) (Table 1). When M185A is introduced into the E183A mutant background (M185A:E183A), pore-forming activity is restored to 44% of that of native PFO at 37°C; however, its pore-forming activity drops off rapidly below 37°C, and so its *E_a_* could not be determined. Therefore, although the M185A mutation was able to restore wild-type or greater activity to the π-stacking mutants, across the temperature range used in our *E_a_* studies, it only partially restored pore-forming activity in the E183A mutant at 37°C and exhibited significantly lower activity at temperatures less than 37°C. This result is consistent with the necessity of the electrostatic interaction to supply additional free energy, along with the π-stacking interaction, to complete the prepore-to-pore transition.

Next, we examined the impact of the M185A mutation on a PFO variant lacking both the π-stacking and electrostatic interactions (M185A:Y181A:E183A mutant). As shown in Table 1, the M185A mutation did not restore activity when both the π-stacking and electrostatic interactions were missing in PFO. This result shows that the free energy provided by both interactions is necessary to overcome the prepore-to-pore transition state energy barrier.

Previous studies showed that the destabilization of the domain 3-domain 1 and 2 interface by mutation of PFO N197 to tryptophan (N197W) restored activity to PFO mutants lacking the E183-K336 electrostatic interaction (14). However, the N197W mutation did not restore activity to the Y181A π-stacking mutant (N197W:Y181A variant) (Table 1). These data suggest that the π-stacking interaction drives a conformational change that is required for the subsequent establishment of the electrostatic interaction, which we have shown destabilizes the interface formed by the domain 3 αHB1 with domains 1 and 2 (14, 15). Hence, the facts that M185 can partially restore activity to PFO lacking the electrostatic interaction but that N197W cannot restore activity to PFO lacking the π-stacking interaction suggests that the π-stacking interaction occurs prior to and is required for the formation of the electrostatic interaction.

### Interacting partners of M185.

To identify the residues that interact with M185, the PFO crystal structure (16) was subjected to analysis using the interaction energy matrix (IEM) software (17, 18), which calculates the energetics of the interaction for each residue in PFO against all other residues in PFO. IEM analysis identified a water-restricted region between the domain 3 core β-sheet and αHB2, which is bordered by residues M185, M222, and Q291 (Fig. 2). This analysis indicated that the M185 side chain has the most favorable (attractive) interaction energies with residues M222, Y187, and Q291 (Table 2). Other residues, such as V221, Q191, S184, and V186, are not considered because their primary interactions are with the backbone of M185, which would not change upon mutation of M185. F294 exhibits a weak side chain interaction with M185 (∼5 kj/mol), which results primarily from weak van der Waals interactions, as its sulfur atom is not positioned to satisfy a sulfur-π interaction (19). However, as described below, F294 plays an important role in this scenario.

**TABLE 2:** Interaction energy matrices for PFO M185, M222, and F294^*a*^

M185 interaction	IE (M185A)	M222 interaction	IE (M222A)	F294 interaction^*b*^	IE (F294A)
Q291	–15 (−1)	F294	–13 (−2)	L281	–20 (−2)
M222	–9 (−2)	L281	–8 (−2)	M222	–9 (−3)
Y187	–8 (−4)	K295	–4 (0)	M185	–3 (0)
V221	–7 (−1)	M185	–3 (−3)		
F294	–5 (0)	I298	–3 (0)		
S184	–4 (−2)	Q291	–2 (0)		

Total IE	–48 (−10)		–33 (−7)		–32 (−5)

a The interaction (attractive) energy (IE) in kilojoules per mole was determined for the side chains of M185, M222, and F294 with all other residues in the structure of PFO (Protein Data Bank accession number 1PFO). The IEs for the alanine substitution mutants are shown in parentheses. IEs of ≤2 kJ/mol for native PFO are not shown. Interactions of residue side chains with the backbone of M185, M222, and F294 and their alanine derivatives are not shown since these will not change upon mutation.

b Only those interactions with core β-sheet residues are shown for F294.

The IEM results were tested by individually mutating residues M222, Q291, and Y187 to alanine and then determining the specific activity of pore formation and the *E_a_* of any mutant that could suppress the loss of the π-stacking interaction (Y181A). Each mutant increased the specific activity of PFO 2- to 3-fold (Table 1). When either Q291A or Y187A was combined with the prepore-trapped Y181A mutation (Q291A:Y181A and Y187A:Y181A), pore-forming activity was not restored (Table 1). Therefore, the loss of these interactions alone with M185 could not explain the ability of M185A to restore activity to Y181A or F318A.

M222A, however, restored pore-forming activity to the Y181A mutants (Table 1). Like M185A, M222A increases the pore-forming activity of PFO >2-fold and lowers its *E_a_* to 18 kcal/mol (Fig. 2). When M222A was combined with Y181A (M222A:Y181A), like M185A, it restored pore-forming activity, and its *E_a_* was determined to be 32 kcal/mol (Fig. 2). Therefore, M185A and M222A restored activity to Y181A, which suggests that they mediate interactions that are necessarily disrupted by the formation of the Y181-F318 π-stacking interaction, and therefore, their interactions contribute to the prepore-to-pore transition state energy barrier.

### Alanine substituting for F294 restores pore-forming activity in the absence of the aromatic π-stacking interaction.

Since M222 was the only interacting partner with the M185 side chain, which restored activity to Y181A, we performed an IEM analysis of its interacting partners. These results show that the M222 side chain has its strongest attractive interactions with F294 in αHB2 and the side chain of the neighboring core β-sheet residue L281. M185, M222, and L281 are arranged next to each other in β-strands 1 to 3 of the core β-sheet, respectively (Table 2 and Fig. 2). When L281 was mutated to alanine (L281A), it nearly eliminated all of its major interactions (Table 2), yet the specific activity of pore formation for PFO was largely unchanged, and it did not restore activity to the Y181A π-stacking mutant (L281A:Y181A) (Table 1).

In contrast to L281A, the mutation of F294 to alanine (F294A) dramatically increases the specific activity of PFO pore formation (>6-fold) and decreases its *E_a_* to 21 kcal/mol (Table 1 and Fig. 2). Interestingly, when F294A was combined with Y181A (F294A:Y181A), we observed almost identical changes in the specific activity of pore formation and its *E_a_* (Table 1 and Fig. 2). These studies suggest that the interactions mediated by the M185-M222-F294 triad at the interface of the αHB2 interface with the core β-sheet (Fig. 2) are disrupted by the formation of the π-stacking interaction. F294 appears to play a special role in this interaction. As indicated above, it decreases the *E_a_* and specific activity of pore formation for PFO to the same extent, whether or not the Y181A mutation is present. In contrast, the M185A and M222A mutations lower the *E_a_* of PFO to a slightly greater extent than F294A, but when they are combined with Y181A, their activation energies are increased to 32 to 38 kcal/mol, compared to 20 kcal/mol for F294A:Y181A. Hence, the F294A mutation eliminates the requirement for the π-stacking interaction in both PFO and PFO^Y181A^, as suggested by their similar activation energies and specific activities of pore formation.

### SDS-AGE analysis of oligomers that restore activity to the Y181A π-stacking mutant.

**FIG 3:**
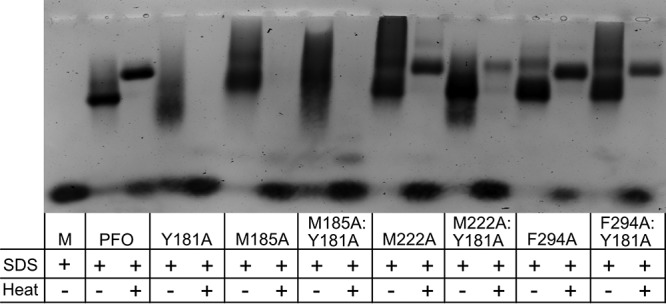
Oligomer formation by PFO and PFO variants. Oligomer formation was assessed by incubating PFO and its derivatives with POPC-cholesterol liposomes for 30 min at 37°C. After incubation, sample buffer was added to the proteoliposomes with or without heating them to 95°C for 3 min. Oligomers were resolved on a 1.5% SDS agarose gel. M, PFO monomer without liposomes and heat.

SDS-agarose gel electrophoresis (SDS-AGE) analysis was performed to determine the size and stability of the oligomers of the M185A, M222A, and F294A PFO mutants and in combination with the Y181A π-stacking mutant (Fig. 3). Previous analyses indicated that Y181A oligomers mostly disassociated in the presence of SDS and were completely dissociated when heated to 95°C but that wild-type PFO oligomers were largely stable in the presence of SDS when heated to 95°C, consistent with our results herein (Fig. 3) (8). The M222A and Y294A mutants generated oligomers that exhibited resistance to dissociation by SDS and heat similar to that observed for PFO, whereas M185A oligomers were resistant to SDS but not SDS and heat. The M222A-Y181A and F294A-Y181A oligomers were less stable in the presence of SDS and heat than PFO, which suggests that the formation of the intermolecular stacking interaction, which appears to remain intact in the native pore structure (Fig. 1), helps stabilize the oligomeric structure. Like the M185A oligomer, the M185A:Y181A mutant oligomers were dissociated by heat and SDS. This suggests that M185 helps stabilize the oligomeric pore. However, the basis for this stabilization is unclear, as M185 does not appear capable of forming any significant side chain-mediated interactions with residues in β-strand 4 of another monomer when analyzed using a PFO pore model based on the pneumolysin cryoEM pore structure (20). Therefore, the basis for the stabilizing influence of M185 on the oligomeric pore structure remains unclear.

### *T_m_*s of PFO and its derivatives.

We have previously shown that the relative stability of the interface between domain 3 and domains 1 and 2 is a major contributor to the melting temperature (*T_m_*) of PFO (14), as this interface is necessarily disrupted to refold αHB1 into TMH1 (11). The *T_m_*s for PFO and its derivatives herein were determined (Table 1). When the M185A, M222A, and F294A mutations are placed into PFO, their *T_m_* decreases 2 to 7°C. The F294A variant exhibits the largest decrease in its *T_m_*: more than 7°C. This is likely due to the loss of F294-mediated interactions with multiple residues (M185, M222, and L281) at the interface of αHB2 with the core β-sheet. Therefore, the interaction of the residues of αHB2 with the core β-sheet contributes to the *T_m_* of PFO, as do those interactions between αHB1 with domains 1 and 2 (14, 15). The decreases in the *T_m_*s of the F294A variant (determined herein) and the N197W variant (determined previously [14]) are also the largest among the single mutants that we have identified. Hence, the interactions between αHB1 and domains 1 and 2, which are mediated largely by networked waters and are disrupted in the N197W variant (15), and between αHB2 and the core β-sheet of domain 3, which is mediated largely by F294, are major forces stabilizing the structure of domain 3 in the monomer. These interactions, however, must be disrupted to extend the α-helical bundles into the membrane-spanning TMHs of the β-barrel pore. The fact that N197W and F294A variants exhibit the largest increase in pore-forming activity and the largest decrease in their *T_m_*s over that of native PFO is consistent with the contribution of the N197- and F294-mediated interactions to the transition state energy barrier for pore formation.

## DISCUSSION

The studies herein show that the intermolecular π-stacking of Y181A with F318A is necessary to disrupt interactions between αHB2 and the core β-sheet mediated by the Met-Met-Phe triad of residues. The disruption of these interactions frees αHB2 to refold into one of the two membrane-spanning β-hairpins that contribute to the formation of the large β-barrel pore for the CDCs. We had previously proposed that the π-stacking interaction ensured that the membrane-spanning β-hairpins remained in the proper register during assembly of the pore (9). However, our data herein show that the π-stacking of these residues is unlikely to function in this manner, as the restoration of pore-forming activity can be achieved in mutants lacking the π-stacking interaction. We further show that the π-stacking interaction precedes but works in tandem with the intermolecular electrostatic interaction, which we have shown (14) drives the disruption of the final contacts between αHB1 and domains 1 and 2. Together, these two intermolecular interactions bring β-strands 1 and 4 of two adjacent monomers into alignment to form backbone hydrogen bonds, thereby flattening the core β-sheet, which simultaneously drives the disruption of the αHB2 contacts with the core β-sheet and the contacts between αHB1 domains 1 and 2. This then allows the αHBs to refold into the membrane-spanning hairpins of the β-barrel pore.

Our studies show that the π-stacking interaction precedes and is required for the formation of the electrostatic interaction. M185A partially restores activity to the electrostatic E183A mutant. However, the N197W mutation, which we previously showed to restore activity to the E183A electrostatic mutant by disrupting the water-stabilized interface between αHB1 and domain 2 (14, 15), does not restore activity to the π-stacking Y181A mutant. Furthermore, M185A does not restore activity to the Y181A:E183A double mutant. These results suggest that the stacking interaction precedes and is necessary for the subsequent formation of the electrostatic interaction. Both interactions, however, are required to drive the disruption of the contacts that prevent the conversion of the αHBs into the TMHs.

**FIG 4:**
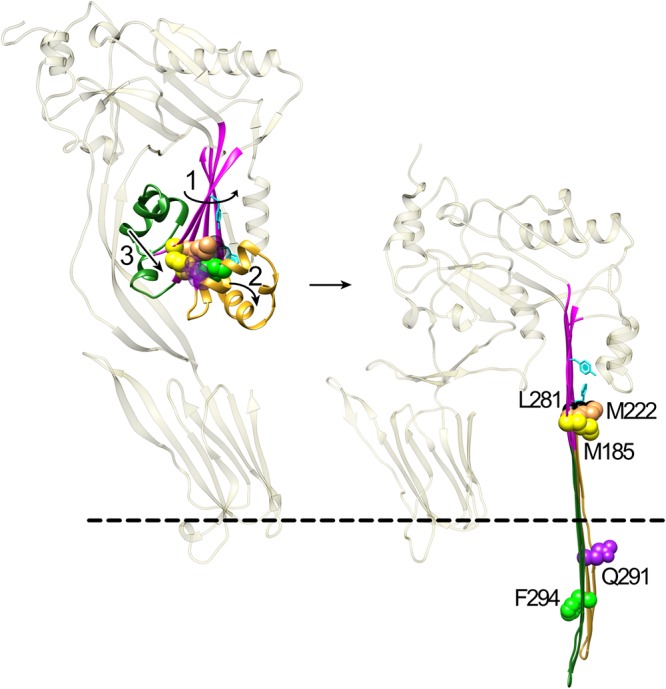
Proposed mechanism by which intermolecular π-stacking and electrostatic interactions drive β-barrel formation. In step 1, the intermolecular π-stacking interaction between Y181 and F318 initially relieves some of the twist in the core β-sheet (magenta), which brings K336 and E183 sufficiently close to form an intermolecular electrostatic interaction. The formation of the electrostatic interaction completes the alignment of β-strands 1 and 4 between two monomers, which flattens the core β-sheet. The progressive flattening of the core β-sheet by the tandem actions of the π-stacking and electrostatic interactions simultaneously disrupts the interactions between F294 and the core β-sheet residues M185, M222, and L281 (step 2) and between αHB1 and domains 1 and 2 (14) (step 3). Both αHB1 and -2, now free of the contacts (or weakened contacts) that maintained their structure, then refold and extend into TMH1 and TMH2, which form the β-barrel pore (2, 10, 11). F294 faces the bilayer, and Q291 faces the pore lumen. M185, M222, and L281 remain lined up in the core β-sheet. F318 is behind L281. The dashed line represents the surface of the bilayer.

How do these two intermolecular interactions drive pore formation? The core β-sheet of the soluble CDC monomer contains a severe twist wherein β-strands 1 and 2 are bent back toward domain 2 and β-strand 4 extends away from domain 2 (Fig. 1A and B). This twist is relieved and the β-sheet flattened in the pore complex to form the upper part of the β-barrel pore (Fig. 1), as predicted from studies of the membrane-spanning β-hairpin backbone hydrogen bond alignment (10) and recently confirmed in the cryoelectron microscopy (cryoEM) structure of the pneumolysin pore (20). The flattening occurs as β-strands 1 and 4 of the adjacent monomers within the prepore complex are brought into alignment with each other to form their backbone hydrogen bonds. We have previously shown that E183 forms an electrostatic interaction with K336 in β-strand 5. This interaction draws β-strands 1 and 4 of adjacent monomers in the prepore together, which weakens the interaction of αHB1 with domains 1 and 2 (14, 15). As indicated above, the π-stacking interaction appears to precede and is necessary for the establishment of the electrostatic interaction. Therefore, the π-stacking interaction begins the process of aligning β-strands 1 and 4, which then brings E183 and K336 sufficiently near each other to form an electrostatic interaction. Their combined interactions align β-strands 1 and 4, enabling the establishment of the interstrand backbone hydrogen bonds, which flattens of the core β-sheet (Fig. 4, step 1). The flattening of the core β-sheet simultaneously results in the disruption/weakening of the interactions between αHB2 and the core β-sheet (as suggested by the lower *T_m_* of the Met-Met-Phe mutants) and between αHB1 and domains 1 and 2 (14, 15) (Fig. 4, steps 2 and 3). This frees the αHBs of their restraints to then refold into the extended β-hairpins that form the β-barrel pore (Fig. 4, right panel).

Why do mutations of M185, M222, or F295 restore activity to mutants that lack the intermolecular π-stacking interaction (i.e., Y181A or F318A) and remain trapped in the prepore state? The structure of domain 3 in the soluble monomer shows that these three residues form interactions with each other that contribute to maintaining the twist in the core β-sheet (Fig. 1D). F294 in αHB2 forms strong interactions with M222 and L281 in β-strands 2 and 3 of the core β-sheet, respectively. M185 in β-strand 1 forms strong interactions with M222 and with Q291 in αHB2. These interlocking interactions help maintain the twist in the core β-sheet and force β-strands 1 and 2 to adopt a structure that bends them back toward domain 2. The bent structure of these β-strands is also facilitated by the interaction of Y187 with the domain 2 residues Y381 and E383 (Fig. 1D), but the loss of these Y187-mediated contacts does not restore activity to the π-stacking Y181A mutant. Likewise, the interactions of M185 with Q291 in αHB2 and L281 with F294 are not key interactions, since their individual mutation to alanine does not restore activity to the π-stacking Y181A mutant. This does not mean that these residues do not play a role in stabilizing the twist in the core β-sheet, but they are not the key residues that play central roles in maintaining the core β-sheet structure. Unlike with these residues, the loss of either M185 or M222 would leave a gap in this set of interlocking residues that would eliminate several key interactions, which likely increases the flexibility in β-strands 1 and 2 of the core β-sheet. The enhanced flexibility of these strands would increase the probability that β-strand 1 partially straightens and begins forming backbone hydrogen bonds with β-strand 4 and bring E183 and K336 sufficiently close to form the electrostatic interaction. These interactions would complete the process of aligning β-strands 1 and 4 and finish disrupting the final interactions of αHB1 and -2 with domain 2 and the core β-sheet, respectively, allowing them to refold into the membrane-spanning β-hairpins.

F294 is the keystone residue in this triad of residues. When M185, M222, or F294 are replaced with alanine, they significantly lower the *E_a_* of pore formation in PFO by 9 to 13 kcal/mol. When M185A and M222A are placed into the Y181A background, they restore pore-forming activity; however, their activation energies increase to 32 to 38 kcal/mol and their specific activities of pore formation are lower than observed for the PFO derivatives that do not contain the Y181A mutation. In contrast, the activation energies and specific activities of pore formation for the F294A and F294A:Y181A mutants are the same. These data show that the π-stacking interaction is irrelevant when the F294-mediated interactions are lost. Hence, F294 plays a major role in locking the core β-sheet into its twisted conformation and restricting the movement of αHB1 (Fig. 1D).

More than 80% of the CDCs maintain the π-stacking aromatics equivalent to those of Y181 and F318 of PFO. In these CDCs, analogs to the M185, M222, and Y294 triad are also highly conserved. Most (∼70%) of these CDCs also maintain a homologous electrostatic pair to E183 and K336 of PFO (14) (Table S1). The conservation of the π-stacking and electrostatic pairs of residues suggests that these CDCs have evolved similar approaches to triggering the conformational changes in domain 3 to form the β-barrel pore. A fraction of CDCs, however, do not retain the equivalent π-stacking and/or electrostatic pairs of residues (Table S1). The results herein and those of Wade et al. (14, 15), however, show that the structure of the CDCs can be easily modified by point mutations, which lower the *E_a_* of pore formation and restore activity to mutants that lack these intermolecular interactions. The principles revealed herein also apply to the membrane attack complex perforin (MACPF) and stonefish toxin families, as they must undergo the same α-helical–to–β-strand structural transitions to form a pore (21–28). These studies show that the stability of the two interfaces that maintain the αHB1 and αHB2 structures are the primary factors that influence the magnitude of the prepore-to-pore transition state energy barrier. We have shown herein that their stability can easily be altered to lower the activation energy of this transition, thereby enabling the α-helical–to–β-strand transition in the absence of the π-stacking and electrostatic interactions. Thus, it is not difficult to envision how the evolution of those CDCs that lack these interactions, and of the MACPF and stonefish family of toxins, can manipulate the stability of these interfaces to drive the α-helical–to–β-strand transition in the absence of the π-stacking and electrostatic interactions used by PFO.

10.1128/mBio.01017-19.1 TABLE S1 Shown are the species with genes for putative or characterized CDCs derived from a PSI Blast (33) search of the GenBank database. Some these CDCs have been characterized, while the majority have not been studied. Download Table S1, DOCX file, 0.02 MB. Copyright © 2019 Burns et al. 2019 Burns et al. This content is distributed under the terms of the Creative Commons Attribution 4.0 International license.

## MATERIALS AND METHODS

### Plasmids, bacterial strains, toxin production, and purification.

The gene for the cysteine-less PFO derivative (PFO^C459A^) was codon optimized for Escherichia coli expression (GenScript) and cloned into pET-15b (Novagen). Protein expressed from this plasmid (pRT30) is referred to as PFO herein, and it exhibits pore-forming activity similar to that of native PFO (11). QuikChange mutagenesis (Stratagene) was used to introduce mutations into PFO, using pRT30 as the template. The DNA of each mutant was sequenced and then analyzed using a CLC sequence viewer. Hexahistidine-tagged, signal peptide-deficient PFO and derivatives were transformed into E. coli Tuner cells for expression (Novagen). Cells were cultured, and the recombinant protein was purified and stored under conditions described previously at −80°C (11). Prior to use, thawed proteins were centrifuged at 14,000 × *g* and assayed for concentration.

### Liposome preparation.

Liposomes that contained a 45:55 mol% ratio of 1-palmitoyl-2-oleoyl-*sn*-glycero-3-phosphocholine (POPC; Avanti Polar Lipids) to cholesterol (Steraloids) were generated with and without encapsulated 5(6)-carboxyfluorescein (CF) (Sigma). Liposomes were made as previously described (11, 29).

### Liposome release assay.

The pore-forming activities of PFO and derivatives were measured by incubating serial dilutions of toxin with 100 μl of a 1:1,000 dilution of CF-containing liposomes in HBS (HEPES buffered saline [20 mM HEPES, pH 7.4, 150 mM NaCl]) for 1 h at 37°C. Samples were read on an Infinite 200 Pro (Tecan) optimized for high-count fluorescein detection. The pore-forming activity (i.e., 50% effective concentration [EC_50_]) of PFO and derivatives on CF liposomes was determined as previously described (29). The relative pore-forming activity is reported as a percentage of PFO activity.

### *E_a_* measurements.

PFO and PFO variants (88 pmol/50 μl) were injected into a 2.0-ml stirred solution of HBS with CF liposomes (10 μl). CF release was monitored using an SLM-8100 photon-counting spectrofluorimeter (SLM Instruments) held at a specific temperature. The excitation wavelength was set to 470 nm, and emission was monitored over time at 515 nm, as previously described (14). The initial velocity (*k*_0_) of pore formation (i.e., the linear portion of the CF release curve) was determined at specific temperatures between 15°C and 35°C. The *E_a_* was then calculated from the equation *E_a_* = –*R* · *m*, where *m* is the slope of the line derived by plotting ln(*k*_0_) versus 1/temperature (°K) and *R* is the gas constant (8.314 J/mol/K).

### SDS-AGE.

SDS-AGE was carried out as previously described (11, 30, 31). PFO was incubated in the presence or absence of POPC-cholesterol liposomes for 30 min at 37°C. SDS sample buffer was added to solubilize samples with or without the addition of heat (95°C) for 3 min. Monomeric and oligomeric complexes were then resolved on a 1.5% SDS agarose gel.
