# Supplementary material for: An Intermolecular π-Stacking Interaction Drives Conformational Changes Necessary to β-Barrel Formation in a Pore-Forming Toxin
Source: mBio. 2019 Jul 2;10(4):e01017-19. doi: 10.1128/mBio.01017-19 (PMC6606804; doi:10.1128/mBio.01017-19)
Supplement: TABLE S1 [file mBio.01017-19-st001.docx]

**Supplementary Material**

**Table S1.**

| **CDCs that retain the Phe-Met-Met triad, the intermolecular electrostatic and π-stacking interactions** (*lack the M185 equivalent) | | |
| --- | --- | --- |
| *Bacillus cereus* | *Clostridium bifermentans* | *Paenibacillus aceti* |
| *Bacillus weihenstephanensis* | *Dorea massiliensis* | *Paenibacillus kribbensis* |
| *Bacillus anthracis* | *Paeniclostridium sordelli* | *Paenibacillus peoriae* |
| *Bacillus thuringensis* | *Romboutsia lituseburensis* | ***Clostridium perfringens (PFO)*** |
| *Viridibacillus arenosi* | *Peptostreptococcaceae bacterium VA2* | *Brevibacillus brevis* |
| *Bacillus toyonensis* | *Romboutsia maritimum* | *Clostridium butyricum* |
| *Viridibacillus arvi* | *Clostridium dakarense* | *Streptococcus canis* |
| *Bacillus mycoides* | *Paenibacillus dendritiformis* | *Streptococcus dysgalactiae subsp.equisimilis* |
| *Clostridium drakei* | *Paenibacillus thiaminolyticus* | *Streptococcus pyogenes* |
| *Lysinibacillus fusiformis* | *Paenibacillus sp.OSY-SE* | *Streptococcus didelphis* |
| *Lysinibacillus sphaericus* | *Paenibacillus uliginis* | *Streptococcus ictaluri* |
| *Bacillus cecembensis* | *Paenibacillus alvei* | *Streptococcus urinalis* |
| *Lysinibacillus xylanilyticus* | *Paenibacillus taiwanensis* | *Clostridium haemolyticum* |
| *Bacillus decisifrondis* | *Paenibacillus macquariensis* | *Clostridium massiliodielmoense* |
| *Lysinibacillus parviboronicapiens* | *Paenibacillus terrigena* | *Clostridium novyi NT* (CDC #2) |
| *Brevibacillus laterosporus* | *Paenibacillus etheri* | *Clostridium tetani* |
| *Clostridium lundense* | *Paenibacillus glacialis* | *Clostridium botulinum* |
| *Clostridium tetanomorphum* | *Paenibacillus elgii* | *Clostridium novyi NT* (CDC #1) |
| *Clostridium argentinense* | *Paenibacillus tyrfis* | *Clostridium mangenotii* |
| *Clostridium hydrogeniformans* | *Paenibacillus assamensis* | *Enterobacter lignolyticus** |
| *Austwickia chelonae** |  |  |
| **CDCs that retain the intermolecular π-stacking interaction** | | |
| *Listeria innocua* | *Listeria monocytogenes* | *Listeria ivanovii* |
| *Listeria seeligeria* | *Acetivibrio ethanolgignens* | *Streptococcus suis* |
| *Oxalobacter formigenes* | *Pedobacter borealis* | *Enterococcus faecalis (marine isolate)* |
| *Fusobacterium necrogenes* | *Fusobacterium perfoetens* |  |
| **CDCs that do not retain the Phe-Met-Met triad, the intermolecular electrostatic and π-stacking interactions** (*species that contain either the pneumolysin (1) or lectinolysin genes (2)) | | |
| *Streptococcus pseudopneumoniae* | *Streptococcus mitis** | *Streptococcus oralis subsp. tigurinus* |
| *Gemella bergeri* | *Gemella cuniculi* | *Lactobacillus iners* |
| *Streptococcus intermedius* | *Bifidobacteriaceae bacterium NR003* | *Gardnerella vaginosis* |
| *Sphingomonas paucimobilis* | *Streptococcus pneumoniae* | *Pedobacter borealis* |
| *Devriesea agamarum* | *Arcanobacterium haemolyticum* | *Arcanobacterium phocae* |
| *Trueperella pyogenes* | *Desulfobulbus propionicus* |  |

1. Whatmore AM, Efstratiou A, Pickerill AP, Broughton K, Woodard G, Sturgeon D, George R, Dowson CG. 2000. Genetic relationships between clinical isolates of *Streptococcus pneumoniae, Streptococcus oralis,* and *Streptococcus mitis*: characterization of "Atypical" pneumococci and organisms allied to *S. mitis* harboring S. pneumoniae virulence factor-encoding genes. Infect Immun 68:1374-82.

2. Farrand S, Hotze E, Friese P, Hollingshead SK, Smith DF, Cummings RD, Dale GL, Tweten RK. 2008. Characterization of a streptococcal cholesterol-dependent cytolysin with a lewis y and b specific lectin domain. Biochemistry 47:7097-107.
